# Greater Ultra-Processed Food Intake during Pregnancy and Postpartum Is Associated with Multiple Aspects of Lower Diet Quality

**DOI:** 10.3390/nu14193933

**Published:** 2022-09-22

**Authors:** Tonja R. Nansel, Jenna R. Cummings, Kyle Burger, Anna Maria Siega-Riz, Leah M. Lipsky

**Affiliations:** 1 Social and Behavioral Sciences Branch, Division of Population Health Research, Eunice Kennedy Shriver National Institute of Child Health and Human Development, 6710B Rockledge Dr., MSC 7004, Bethesda, MD 20892, USA; 2 Gillings School of Global Public Health, University of North Carolina Chapel Hill, 2204 McGavran-Greenberg Hall, CB# 7461, Chapel Hill, NC 27599, USA; 3 Departments of Nutrition and Biostatistics and Epidemiology, School of Public Health and Health Sciences, University of Massachusetts, 109 Arnold House, 715 Pleasant St., Amherst, MA 01003, USA

**Keywords:** ultra-processed food, diet quality, pregnancy, postpartum

## Abstract

Low diet quality during pregnancy and postpartum is associated with numerous adverse maternal and infant health outcomes. This study examined relations of ultra-processed food intake with diet quality during pregnancy and postpartum. Using data from 24-h recalls, ultra-processed food intake was operationalized as percent energy intake from NOVA-classified ultra-processed foods; diet quality was measured using Healthy Eating Index 2015 (HEI) total and component scores. Pearson correlations examined associations of ultra-processed food intake with HEI total and component scores, and food group intake was compared across four levels of ultra-processed food intake. On average, ultra-processed food comprised 52.6 ± 15.1% (mean ± SD) of energy intake in pregnancy and 50.6 ± 16.6% in postpartum. Ultra-processed food intake was inversely correlated with HEI total and 8 of 13 component scores. Compared to participants with the highest ultra-processed food intake (≥60% energy), those with the lowest ultra-processed food intake (<40% energy) had a 17.6-point higher HEI total score and consumed 2–3 times more fruit, vegetables, and seafood and plant proteins, and 1½ times more total protein. Additionally, they consumed 2/3 as much refined grains and 1/2 as much added sugar. Greater ultra-processed food intake was associated with lower diet quality across most HEI components. Reducing ultra-processed food intake may broadly improve adherence to dietary guidelines in pregnant and postpartum populations.

## 1. Introduction

The importance of nutrition during pregnancy for supporting optimal maternal and child health is well-established and recognized by healthcare professionals [1,2,3]. However, dietary intake during pregnancy is typified by inadequate consumption of vegetables, fruit, and whole grains, as well as excessive intake of empty calories, fat, and sodium [4,5,6]. Limited evidence suggests diet quality is similarly low in the postpartum period [7,8]. Given associations of low maternal diet quality with a wide range of adverse outcomes for both mother and infant [9,10,11,12,13], there is a critical need to identify modifiable intervention targets.

Ultra-processed foods, that is, food products that are created from substances extracted from foods or derived from food constituents [14], contribute on average greater than half of energy intake in the general U.S. population [15]. Consistent evidence across countries indicates that ultra-processed food intake is associated with select nutrients, including lower intake of fiber [16,17,18,19,20,21,22,23]; higher total and saturated fat [16,17,18,19,20,23]; and higher free, added, or total sugar [16,17,20,21,22,23,24,25] in general population samples. Additionally, four studies have demonstrated an inverse relation of ultra-processed foods with overall diet quality [18,26,27,28]. Consumption of ultra-processed foods may adversely affect overall diet quality via excess intake of highly processed discretionary foods and also by displacing intake of unprocessed, whole plant foods; one study found that greater consumption of ultra-processed food was related to greater intake of sugar-sweetened beverages and processed meat, as well as lower intake of fruits/vegetables, nuts/seeds/legumes, and fish in a general population sample [26]. Understanding the impact of ultra-processed food consumption on intake across healthful and discretionary food groups is important for determining effective approaches to improve diet quality given the pervasiveness of ultra-processed foods in the food environment.

The importance of pregnancy and postpartum diet quality for fostering maternal and child health and the ubiquitous presence of ultra-processed foods in the environment are well-established; yet, the association of ultra-processed food intake with pregnancy and postpartum diet quality is largely unexamined. One study investigated relations of ultra-processed food intake with nutrient intake in pregnant women in Brazil, finding inverse associations with intake adequacy of several individual nutrients and with consumption of traditional foods including rice, beans, fruits, and vegetables [29]. However, this study did not examine associations with overall diet quality and may have limited generalizability to countries with high ultra-processed food intake given the low average intake of ultra-processed foods (22% of energy intake) in the sample. Additionally, no studies have investigated ultra-processed food intake, or the relations of ultra-processed food intake with diet quality, during postpartum. To address these knowledge gaps, this study examined associations of ultra-processed food intake with overall diet quality and diet quality components during pregnancy and postpartum in a U.S. sample.

## 2. Methods

### 2.1. Design, Participants, and Procedures

The Pregnancy Eating Attributes Study (PEAS) was a prospective observational study examining eating-related behaviors from the first trimester of pregnancy through one-year postpartum. Participants were enrolled at ≤12 weeks gestation from two university-based obstetrics clinics in Chapel Hill, North Carolina from November 2014 through October 2016; data collection was completed in June 2018 [30]. Inclusion criteria included the following: ≤12 weeks gestation at enrollment; body mass index ≥18.5 kg/m^2^; age ≥18 and <45 at screening; uncomplicated singleton pregnancy anticipated; access to internet with email; able to complete self-report assessments in English; intention to deliver at the University of North Carolina Women’s Hospital; plan to remain in the geographical vicinity of the clinical site for one year following delivery; and willing to provide informed consent for participation and assent for the baby’s participation. Exclusion criteria included the following: multiple gestations; participant-reported eating disorder; pre-existing diabetes; any medical condition contraindicating participation in the study, such as chronic illnesses or use of medication that could affect diet or weight; and psychosocial condition contraindicating participation in the study. The primary study aims were to examine the roles of reward-related eating, self-control, and home food availability on dietary intake and weight change during pregnancy and postpartum. Power analyses to determine the sample size are described in the report on primary study aims [30]. 

Research staff screened clinical appointment data to identify potentially eligible patients, then verified eligibility at the time of the clinical appointment and obtained signed informed consent from persons choosing to participate in the study. Study visits were conducted once each trimester, and postpartum at 4–6 weeks, 6 months, and 12 months. Self-report measures including dietary recalls were completed online within designated study visit windows. The study was conducted in accordance with the Declaration of Helsinki, and the protocol was approved by the University of North Carolina Institutional Review Board.

### 2.2. Measures

Dietary intake was assessed using a 24 h recall at each study visit window obtained through the National Cancer Institute’s Automated Self-Administered 24-h Recall (ASA24). The ASA24 uses an online interface in which participants delineate all foods consumed for the specified time period. They are prompted to indicate information on food preparation, brands, portion size, and additions. Study staff provided participants with written information on how to access and use the program and also assisted them if they experienced difficulty using the program. Research staff at the University of North Carolina Nutrition and Obesity Research Core then reviewed the data to identify and corrected implausible entries (e.g., food items with implausible energy, fat or weight) and missing food or nutrient values and quantities. The ASA24 assigns food codes from the U.S. Department of Agriculture (USDA) Food and Nutrient Database for Dietary Surveys (FNDDS) to the participant-reported food items and outputs estimates of macronutrient, micronutrient, food categories, and USDA Food Patterns Equivalents Database food groups. The ASA24 has shown strong validity relative to the interviewer-administered automated multiple pass method [31,32]. Dietary records with daily energy intakes of <600 kcal (36 of 1883 records, 1.9%) or >4500 kcal (21 of 1883 records, 1.1%) were reviewed by the investigators for plausibility. All records with intakes <600 kcal were deemed to be likely incomplete and excluded from analysis. Those with intakes of >4500 kcal were determined to reflect plausible intake and were retained.

Ultra-processed food intake was estimated using the NOVA system (not an acronym). NOVA is a classification system developed by researchers at the University of São Paulo that categorizes foods and beverages into four groups based on the degree of processing [14]. Unprocessed or minimally processed foods are those which remain in their original natural form or which have been altered only by removal of inedible or unwanted parts, crushing, grinding, fractioning, refrigerating, freezing, drying, roasting, boiling, pasteurizing, placing in containers, or vacuum packaging (e.g., fresh and frozen fruit, vegetables, and meat; fresh or pasteurized milk). Processed culinary ingredients are substances that are obtained directly from unprocessed/minimally processed foods by pressing, centrifuging, refining, extracting, or mining (e.g., vegetable oils; butter and lard; sugar and molasses). Processed foods are food products made by adding processed culinary ingredients to unprocessed/minimally processed foods, using canning, bottling, or non-alcoholic fermentation (e.g., salted canned vegetables; fruit preserves; salted nuts; sweetened dried fruit). Ultra-processed foods are food products that are created from a series of industrial processes including chemical modification (e.g., hydrolysis), whole food fractioning, additions for palatability (e.g., colors, flavors, emulsifiers), assembly (e.g., pre-frying), and packaging with synthetic materials (e.g., ‘instant’ foods; ready-to-heat pre-prepared pies, pasta, and pizza; mass-produced packaged breads; reconstituted meats; sweet or savory packaged snacks; confectionery desserts; sweetened drinks). Standardized Stata (College Station, TX, USA) code available upon request from the University of São Paulo group was used to assign NOVA categories to ASA24 food codes. Food codes indicating a homemade recipe were classified using the underlying ingredient codes and correspondent nutrition information from the FNDDS [33]. Percent of daily energy intake from ultra-processed foods was calculated separately for pregnancy (*n* = 365) and postpartum (*n* = 266) by dividing the average total daily energy from ultra-processed foods by the average total daily energy intake across all dietary recalls for each time period. 

The Healthy Eating Index 2015 (HEI) total and component scores [34] were used to indicate overall and specific aspects of diet quality. The HEI indicates degree of conformance to the 2015 US Dietary Guidelines for Americans [35] and is calculated from the ASA24 dietary intake data not including supplements. HEI scores include 9 adequacy components (total fruit, whole fruit, total vegetables, greens and beans, whole grains, dairy, total protein, seafood and plant proteins, fatty acids) and 4 moderation components (refined grains, sodium, added sugars, and saturated fats). All 13 component scores are summed to calculate the HEI total score, which ranges from 0–100. Scores are calculated on a per-1000 kcal or percent of kcal basis for comparability across persons having varying energy requirements. HEI total and component scores were calculated across pregnancy and across postpartum using all dietary recalls for each time period.

Demographic information including education, household income, household composition, race, ethnicity, and marital status were reported by participants at the initial assessment. Family income and household size were used to calculate income-to-poverty ratio [36]. Values were categorized as ≤1.85 (the threshold for eligibility for Special Supplemental Nutrition program for Women, Infants, and Children), 1.86–4.0, and ≥4.0. Participant age and parity were obtained from the electronic medical record; age was categorized by tertiles. Measured height and weight obtained at the initial clinic visit were used to calculate body mass index as kg/m^2^ and categorized as 18.0–24.9, 25.0–29.9, and ≥30.0.

### 2.3. Analysis

Differences in ultra-processed food intake by participant characteristics were examined using analysis of variance or Kruskal–Wallis test when the variance homogeneity assumption was not met. Given expected correlations among sociodemographic characteristics, regression analysis was used to examine associations of ultra-processed food intake with the set of participant characteristics demonstrating significant bivariate associations. Pearson correlations were used to examine associations of ultra-processed food intake with HEI total and component scores. HEI scores and food group intake density values were then compared between participants consuming <40.0, 40.0 to 49.9, 50.0 to 59.9, and ≥60% energy intake from ultra-processed foods; these categories were selected to create consistent cut-points across pregnancy and postpartum based on the sample distribution (roughly quartiles). Food group intakes are provided as density values (e.g., cup or ounce equivalents per 1000 kcal) for ease of interpretation of the resulting mean values.

## 3. Results

Of 458 participants enrolled, 91 (20%) withdrew prior to delivery and 46 (10%) withdrew during the one-year postpartum period; 383 provided dietary intake data during pregnancy and/or postpartum. Mean ± SD baseline age was 30.8 ± 4.6, and 92% were married or living with their partner. Approximately half of the sample were nulliparous, three-quarters had a bachelor’s degree or above, 22% had income ≤185% of the poverty level, and 31% identified as racial or ethnic minority (Table 1). Approximately half had normal weight, while one-quarter each had overweight and obesity. In bivariate analyses, ultra-processed food intake was higher among participants <29 years of age versus older age groups, those with less than a bachelor’s degree versus participants with higher education attainment, those with an income-to-poverty ratio ≤1.85 versus above, those married versus those divorced/separated/widowed/single, and those with overweight or obesity compared to those having normal weight. Ultra-processed food intake was lower among Asian participants than White, Black, and Hispanic participants. In the regression model entering age, education, poverty-to-income ratio, marital status, and body mass index simultaneously, only age (standardized B = −0.13, *p* = 0.03) and education (standardized B = −0.25, *p* < 0.001) were significantly associated with ultra-processed food intake. (Race/ethnicity was not included in the model given the small cell size of the one race/ethnicity that demonstrated significant differences from others.)

Mean ± SD ultra-processed food was 52.6 ± 15.1% of energy intake in pregnancy and 50.6 ± 16.6% in postpartum. In both pregnancy and postpartum, ultra-processed food intake was inversely correlated with HEI total score and with 8 of the 13 component scores, including 6 of the 9 adequacy components and 2 of the 4 moderation components (Table 2).

HEI total scores and food group intake density by categories of ultra-processed food intake are detailed in Table 3. Compared to those consuming ≥60% of energy intake from ultra-processed food, participants consuming <40% energy from ultra-processed food had a 17.6-point higher total HEI score and consumed nearly 2 times more total vegetables, 3 times more greens and beans, 2–3 times more total and whole fruit, about 1½ times more total protein, and about 3 times more seafood and plant protein. Conversely, those consuming ≥60% of energy intake from ultra-processed food consumed about 1½ times more refined grains and more than twice as much added sugar as those consuming ≥60% of energy intake from ultra-processed food.

## 4. Discussion

Ultra-processed food comprised over half of energy intake during both pregnancy and postpartum in this North Carolina sample, similar to the general U.S. population, in which ultra-processed food contributes 57% of total energy intake [15]. Ultra-processed food intake was greater in younger participants and those having less than a bachelor’s degree; associations of ultra-processed food intake with income-poverty ratio, marital status, and body mass index were not significant after adjusting for other participant characteristics. Consistent with previous research in general population samples demonstrating an inverse association of ultra-processed food intake with several indicators of overall diet quality [18,26,27,28], intake of ultra-processed food in pregnancy and postpartum was inversely associated with the HEI total score and with 8 of 13 HEI component scores. Differences in diet quality between those consuming <40% energy intake from ultra-processed food and those consuming ≥60% energy intake from ultra-processed food were of considerable magnitude, with more than a 17-point difference in the HEI total score and a 1.5- to 3-fold difference in intake of eight component food groups.

Ultra-processed food intake was inversely associated with intake of both adequacy and moderation HEI component scores. Greater intake of ultra-processed food was associated with lower intake of total vegetables, greens and beans, total fruit, whole fruit, total protein, and seafood and plant protein. Several of these food groups provide nutrients that are critically important during pregnancy, including protein (total protein, seafood and plant protein); iron, vitamin B6, and zinc (protein food groups, greens and beans, fruit), folate (greens and beans, fruit, seafood and plant protein), vitamin E (plant protein, fruit, vegetables), and magnesium (greens and beans, plant protein) [2]. Greater intake of ultra-processed food was also associated with greater intake of refined grains and added sugar, which provide minimal nutritional value and contribute to excess energy intake. Insufficient adequacy components and excess moderation components both represent risk to the developing fetus. As described by the developmental origins of health and disease hypothesis, inadequate intake of key nutrients, as well as excess energy intake during fetal development, may result in reprogramming within fetal tissues that predisposes the offspring to the development of future chronic disease [37]. The absence of an association of ultra-processed food intake with saturated fat intake is contrary to previous research in general population samples [16,17,18,19,20,21,23], and may be attributable to widespread reformulation of processed food products to avoid hydrogenated fats.

The relations of greater intake of ultra-processed food with lower intake of fruits and vegetables observed herein suggests that ultra-processed foods, which may be perceived as more palatable or convenient, displace intake of whole plant foods. Findings from rodent models indicate that exposure to highly palatable foods leads to rapid suppression of motivation and preference for and intake of standard chow [38], suggesting a mechanism by which widespread exposure to highly palatable, ultra-processed foods may displace consumption of unprocessed foods in free-living humans. In response to systematic findings that ultra-processed food intake is associated with obesity and chronic disease [39], previous authors have discussed the merits of public health policies targeting reformulation versus reduction of ultra-processed food [40]. Reformulation of ultra-processed food could improve diet quality through reduction of added sugar and refined grains, and by addition of fruit, vegetables, and plant protein where feasible (e.g., to packaged meals). However, to the extent that highly palatable ultra-processed foods displace intake of whole plant and protein foods, reformulating ultra-processed food to reduce undesirable nutrients is unlikely to be sufficient to negate the adverse impact of ultra-processed food on overall diet quality.

Given the critical role of nutrition during pregnancy and postpartum for maternal and child health [3] and emerging evidence that ultra-processed food is associated with adverse pregnancy and infant outcomes [41,42,43,44,45], findings from this study suggest that dietary guidance to reduce ultra-processed food intake may represent a singular intervention target with broad impact on adherence to dietary guidelines. Considering health care provider concerns that time constraints limit their ability to provide nutritional guidance during routine pregnancy visits [46], brief messages with broad-ranging impact may be especially valuable. Interventions promoting intake of minimally processed foods in Brazilian pregnant persons have resulted in reduced intake of ultra-processed foods [47] and reduced risk of gestational weight gain [48], providing evidence for the feasibility of such an approach Additionally, nutrition educators may assist pregnant persons in selecting the most nutritious options when they do choose to consume ultra-processed food, taking into consideration the resources and foods available to their clients. 

Findings should be interpreted with consideration of the study’s strengths and limitations. While all methods of self-reported dietary intake assessment have some measurement error due to faulty recall, over-reporting, and under-reporting, 24-h dietary recalls are the least biased self-report measure of dietary intake [49]. NOVA classifications were determined using standardized statistical code and disaggregating foods to their underlying ingredients [33] to ensure accurate application of the classification system. The sample size was relatively large with a wide range of diet quality represented and had similar sociodemographic characteristics to the geographic area of the study site. This county in North Carolina has somewhat higher median household income ($74,803) than the overall U.S. population ($64,994), but with a similar percent of persons in poverty (10% vs. 11% nationally). The area is also more highly educated (61% vs. 33% having a bachelor’s degree or higher) and has a higher proportion of non-Hispanic White race/ethnicity (69% vs. 59% White) [50,51]. Future research in samples disproportionally affected by low resources or low access to grocery outlets would be informative, as the adverse impact of ultra-processed foods on diet quality could be even more pronounced in populations lacking access to a wide range of foods.

In conclusion, in this U.S. sample followed from early pregnancy through one-year postpartum, ultra-processed food accounted for more than half of energy intake and was associated with lower diet quality across most HEI components. Intervention studies testing the impact of approaches targeting reduction in ultra-processed food intake on diet quality are needed to determine their efficacy in improving adherence to dietary guidelines in pregnant populations.

## Figures and Tables

**Table 1:** Sample characteristics and association with ultra-processed food intake.

	Sample Distribution *N* (%)	Percent of Energy Intake from Ultra-Processed Foods in Pregnancy Mean ± SD *
Age		
<29.00	110 (28.7)	58.0 ± 14.9 ^a^
29.00–32.99	132 (34.5)	50.8 ± 15.2 ^b^
≥33.00	141 (36.8)	50.2 ± 14.3 ^b^
Education		
High school or less	29 (8.3)	59.3 ± 16.2 ^a^
Associates/some college	65 (18.5)	59.5 ± 16.9 ^a^
Bachelors	106 (30.2)	52.4 ± 12.9 ^b^
Advanced degree	151 (43.0)	48.6 ± 13.9 ^b^
Income poverty ratio		
≤1.85	77 (22.2)	57.4 ± 17.6 ^a^
1.86–4.00	84 (24.2)	52.7 ± 15.6 ^b^
≥4.00	186 (53.6)	51.0 ± 13.2 ^b^
Race/ethnicity		
White, non-Hispanic	251 (68.8)	52.5 ± 13.8 ^a^
Black, non-Hispanic	53 (14.5)	56.5 ± 17.7 ^a^
Asian	17 (4.7)	39.9 ± 16.2 ^b^
Hispanic	29 (7.9)	53.9 ± 16.3 ^a^
Multi-race & other	15 (4.1)	50.7 ± 17.7 ^ab^
Marital status		
Married/living with partner	322 (91.7)	51.9 ± 14.8 ^a^
Divorced/separated/widowed/single	29 (8.3)	61.2 ± 14.8 ^b^
Parity		
Nulliparous	188 (49.1)	52.3 ± 15.1
Parous	195 (50.9)	52.8 ± 15.2
Body mass index		
18.0–24.9	191 (49.9)	50.5 ± 14.2 ^a^
25.0–29.9	99 (25.8)	54.0 ± 14.5 ^b^
≥30.0	93 (24.3)	55.6 ± 17.2 ^b^

*N* = 383 participants with dietary intake data during pregnancy and/or postpartum. Demographic data missing for 32 participants on education, 36 participants on income, 18 participants on race/ethnicity, and 32 participants on marital status. * Group differences tested using analysis of variance (age, education, marital status, parity, and body mass index) or Kruskal–Wallis test (income poverty ratio and race/ethnicity). Different superscript letters indicate statistically significant differences between groups at *p* < 0.05.

**Table 2:** Pearson correlations of ultra-processed food intake with Healthy Eating Index 2015 (HEI) total and component scores.

	Percent of Energy Intake from Ultra-Processed Foods	
	Pregnancy	Postpartum
HEI total	−0.55 **	−0.52 **
Total vegetables	−0.34 **	−0.40 **
Greens & beans	−0.36 **	−0.28 **
Total fruit	−0.41 **	−0.26 **
Whole fruit	−0.35 **	−0.28 **
Whole grains	−0.16	−0.16
Dairy	−0.09	0.06
Total protein foods	−0.24 **	−0.37 **
Seafood & plant protein	−0.43 **	−0.40 **
Fatty acid ratio	−0.09	−0.18
Sodium	0.02	−0.05
Refined grains	−0.35 **	−0.38 **
Saturated fat	−0.10	−0.16
Added sugar	−0.50 **	−0.49 **

** *p* < 0.001; Sidak-adjusted *p*-values.

**Table 3:** Healthy Eating Index 2015 (HEI) total score and food group intake density by category of ultra-processed food intake.

	Percent of Energy Intake from Ultra-Processed Foods Mean (95% Confidence Interval)			
	<40.0 ^a^	40.0–49.9 ^a^	50.0–59.9 ^a^	≥60 ^a^
HEI total score				
Pregnancy	66.3 (63.6, 69.0)	62.6 (60.6, 64.6)	57.2 (55.1, 59.4)	48.8 (46.7, 50.9)
Postpartum	66.0 (63.1, 68.9)	62.6 (59.5, 65.8)	57.2 (54.1, 60.2)	48.5 (46.0, 51.0)
Total vegetables ^b^				
Pregnancy	1.15 (1.00, 1.30)	1.00 (0.91, 1.09)	0.99 (0.90, 1.08)	0.67 (0.60, 0.73)
Postpartum	1.38 (1.20, 1.57)	1.13 (1.00, 1.26)	0.93 (0.82, 1.04)	0.73 (0.64, 0.82)
Greens & beans ^b^				
Pregnancy	0.33 (0.26, 0.40)	0.27 (0.23, 0.31)	0.24 (0.18, 0.29)	0.10 (0.08, 0.12)
Postpartum	0.39 (0.30, 0.48)	0.32 (0.23, 0.41)	0.24 (0.18, 0.30)	0.13 (0.09, 0.17)
Total fruit ^b^				
Pregnancy	1.23 (1.02, 1.45)	0.84 (0.73, 0.94)	0.67 (0.57, 0.76)	0.45 (0.38, 0.52)
Postpartum	0.71 (0.57, 0.86)	0.62 (0.48, 0.76)	0.53 (0.43, 0.64)	0.35 (0.26, 0.44)
Whole fruit ^b^				
Pregnancy	0.97 (0.77, 1.16)	0.71 (0.60, 0.81)	0.56 (0.46, 0.65)	0.31 (0.25, 0.37)
Postpartum	0.62 (0.48, 0.75)	0.50 (0.38, 0.62)	0.45 (0.35, 0.55)	0.24 (0.17, 0.31)
Whole grains ^c^				
Pregnancy	0.64 (0.47, 0.81)	0.56 (0.47, 0.66)	0.55 (0.45, 0.64)	0.39 (0.32, 0.47)
Postpartum	0.68 (0.52, 0.85)	0.77 (0.56, 0.99)	0.48 (0.37, 0.59)	0.45 (0.35, 0.55)
Dairy ^b^				
Pregnancy	1.06 (0.92, 1.20)	1.03 (0.95, 1.12)	0.99 (0.91, 1.08)	0.90 (0.82, 0.97)
Postpartum	0.82 (0.67, 0.97)	0.96 (0.81, 1.11)	0.97 (0.86, 1.08)	0.83 (0.71, 0.95)
Total protein foods ^c^				
Pregnancy	3.78 (3.38, 4.18)	3.26 (3.04, 3.47)	2.89 (2.63, 3.15)	2.43 (2.25, 2.61)
Postpartum	4.54 (4.17, 4.90)	3.68 (3.33, 4.02)	3.21 (2.90, 3.53)	2.88 (2.56, 3.21)
Seafood & plant protein ^c^				
Pregnancy	1.56 (1.25, 1.85)	1.19 (1.04, 1.35)	0.96 (0.72, 1.20)	0.50 (0.37, 0.62)
Postpartum	1.92 (1.62, 2.22)	1.47 (1.19, 1.75)	1.13 (0.87, 1.39)	0.66 (0.46, 0.87)
Fatty acid ratio ^d^				
Pregnancy	1.86 (1.73, 1.99)	1.70 (1.62, 1.79)	1.69 (1.61, 1.78)	1.75 (1.67, 1.83)
Postpartum	2.09 (1.92, 2.27)	1.93 (1.74, 2.11)	1.76 (1.64, 1.87)	1.80 (1.69, 1.91)
Sodium ^e^				
Pregnancy	1.78 (1.68, 1.87)	1.77 (1.70, 1.84)	1.79 (1.72, 1.87)	1.76 (1.70, 1.81)
Postpartum	1.86 (1.74, 1.98)	1.83 (1.74, 1.91)	1.78 (1.71, 1.85)	1.88 (1.78, 1.98)
Refined grains ^c^				
Pregnancy	2.18 (1.94, 2.41)	2.47 (2.28, 2.66)	2.75 (2.57, 2.93)	3.18 (3.01, 3.36)
Postpartum	1.93 (1.62, 2.24)	2.27 (2.04, 2.50)	2.78 (2.52, 3.05)	2.97 (2.72, 3.22)
Saturated fat ^f^				
Pregnancy	11.5 (10.7, 12.4)	12.7 (12.2, 13.3)	12.9 (12.4, 13.4)	12.6 (12.0, 13.1)
Postpartum	12.2 (11.0, 13.4)	11.7 (10.8, 12.5)	12.7 (12.0, 13.4)	12.6 (11.9, 13.2)
Added sugar ^f^				
Pregnancy	6.5 (5.5, 7.6)	9.2 (8.5, 9.9)	10.7 (10.0, 11.5)	13.4 (12.3, 14.6)
Postpartum	5.9 (5.0, 6.8)	8.4 (7.6, 9.2)	10.3 (9.4, 11.2)	12.8 (11.3, 14.3)

^a^ Sample distribution in pregnancy: <40.0 *n* = 65; 40.0–49.9 *n* = 95; 50.0–59.9 *n* = 101; ≥60 *n* = 104. Sample distribution in postpartum: <40.0 *n* = 68; 40.0–49.9 *n* = 61; 50.0–59.9 *n* = 62; ≥60 *n* = 75. ^b^ cup equivalents/1000 kcal. ^c^ oz equivalents/1000 kcal. ^d^ (PUFAs + MUFAs)/SFAs. ^e^ g/1000 kcal. ^f^ % of energy intake.

## Data Availability

Data described in the manuscript, code book, and analytic code will be made available upon request pending application and approval.
